# Dual-Ionization SPME-GC–HRMS Metabolomic Profiling of Broccoli Volatiles for the Construction of a Broccoli Metabolic Database

**DOI:** 10.3390/molecules30183781

**Published:** 2025-09-17

**Authors:** Chenxue Song, Meijia Yan, Sue Lin, Junliang Li, Huixi Zou, Zhiwei Hu, Xiufeng Yan

**Affiliations:** National and Local Joint Engineering Research Center of Ecological Treatment Technology for Urban Water Pollution, Zhejiang Provincial Key Laboratory of Water Ecological Environment Treatment and Resource Protection, College of Life and Environmental Science, Wenzhou University, Wenzhou 325035, China; songchenxue8625@163.com (C.S.); yanmj980703@163.com (M.Y.); iamkari@163.com (S.L.); lijunliang@wzu.edu.cn (J.L.); zjuzhx@wzu.edu.cn (H.Z.)

**Keywords:** broccoli metabolic database, dual-ionization, GC–HRMS

## Abstract

Volatile organic compounds (VOCs) play critical roles in broccoli’s sensory attributes, defense mechanisms, and ecological interactions, yet comprehensive profiling of its volatilome remains limited. This study aimed to construct a robust and inclusive volatile metabolite database for broccoli using advanced analytical techniques. A pooled sample comprising florets from 191 cultivars was prepared to capture broad chemical diversity and analyzed using solid-phase microextraction–gas chromatography–high-resolution mass spectrometry (SPME-GC-HRMS) under dual ionization modes: electron ionization (EI) and chemical ionization (CI). A total of 206 VOCs spanning nine chemical classes were detected, with 37 compounds further confirmed through synchronized CI analysis. To validate the database, broccoli florets from seven distinct cultivars were analyzed using the same workflow. Of the 206 compounds, 187 (90.78%) were detected in at least one cultivar, while 38 were consistently found across all samples, indicating a conserved core volatilome. Principal component analysis revealed distinct VOC profiles among cultivars, and freeze-dried samples were found suitable for reproducible large-scale analysis. This study demonstrates that a pooled-sample strategy coupled with dual-ionization GC-HRMS provides comprehensive and reliable VOC coverage. The resulting database offers a valuable resource for metabolomics studies in *Brassica*, with applications in cultivar differentiation, flavor research, and environmental response profiling.

## 1. Introduction

Volatile metabolomics plays a pivotal role in understanding the biochemical composition, sensory attributes, and physiological responses of vegetables. Volatile organic compounds (VOCs) contribute not only to flavor and aroma but also serve as biomarkers for plant stress responses, developmental stages, and post-harvest quality [1,2]. Among the available analytical platforms, gas chromatography–mass spectrometry (GC-MS) has emerged as the gold standard for VOC profiling due to its high sensitivity, reproducibility, and efficient compound separation [3]. In particular, solid-phase microextraction (SPME) coupled with GC-MS provides a non-destructive, solvent-free sampling approach and is widely employed in plant metabolomics studies [4,5]. The advent of high-resolution mass spectrometry (HRMS), such as GC-Orbitrap-MS, has further enhanced compound identification by enabling accurate mass measurements and improved structural elucidation [6,7]. Recent studies have shown that VOC profiles in Brassicaceae vegetables, including broccoli and cabbage, vary substantially among cultivars, influenced by both genetic background and environmental conditions [8,9]. Consequently, volatile metabolomics using GC-MS not only supports cultivar differentiation but also informs breeding strategies aimed at improving flavor, nutritional quality, and stress resilience.

Electron ionization (EI) remains the most widely used ionization technique in GC-MS analysis due to its high reproducibility, standardized spectral libraries, and rich fragmentation patterns that facilitate compound identification. However, the extensive fragmentation associated with EI frequently results in a low abundance or complete absence of the molecular ion, which can hinder accurate molecular weight determination and the identification of unknown compounds [10]. To overcome this limitation, chemical ionization (CI) offers a softer alternative that generates less fragmentation and typically preserves the molecular ion, thereby providing complementary structural information [11,12]. CI spectra are highly dependent on the choice of reagent gas (e.g., methane, isobutane), which influences ion–molecule reaction pathways and can enhance the detection of labile or thermally sensitive compounds [13]. The combined use of EI and CI modes enables a more comprehensive characterization of volatile compounds, particularly in complex plant matrices. While EI delivers detailed fragmentation useful for spectral library matching, CI facilitates accurate molecular mass determination, thereby improving confidence in compound annotation [12]. Integrating both ionization techniques into GC-MS workflows significantly enhances the coverage and reliability of volatile metabolite identification.

Broccoli (*Brassica oleracea* L. var. *italica* Plenck) is a widely consumed cruciferous vegetable, prized for both its culinary versatility and its rich nutritional and phytochemical profile. It is an excellent source of essential vitamins (particularly C, K, and folate), minerals, dietary fiber, and bioactive compounds such as glucosinolates and flavonoids [14]. Beyond its nutritional value, broccoli has also emerged as a model species in plant science due to its well-characterized glucosinolate–myrosinase system [15,16], its role as a nutritionally relevant crop [17], and its suitability for studies of secondary metabolism [18,19], flavor biochemistry [20], stress responses [21], harvest physiology [22], and phytochemical profiling [23]. Collectively, these attributes make broccoli a tractable and informative system for both basic and applied plant research. In recent years, growing attention has been directed toward its metabolomic and VOC profiles to better understand sensory attributes and postharvest quality. For example, exogenous application of methyl jasmonate has been shown to stimulate glucosinolate biosynthesis and enhance the accumulation of sulforaphane and flavonoids [24]. Moreover, primary metabolite profiling has highlighted the role of amino acids in influencing taste, thereby informing breeding strategies aimed at flavor improvement [9]. Analytical platforms such as GC-MS have been effectively applied to assess freshness and characterize VOC emissions in fresh-cut broccoli [25]. However, despite these advances, comprehensive characterization of the full spectrum of broccoli’s volatile metabolome remains limited, underscoring the need for more integrative and high-resolution approaches.

The construction of a comprehensive metabolic database for broccoli volatile compounds is essential for advancing research in plant science, food quality, and human nutrition. Such databases facilitate the accurate identification and systematic cataloging of the diverse VOCs that contribute to broccoli’s distinctive flavor, aroma, and health-promoting properties [25,26]. When combined with high-resolution analytical techniques such as GC–HRMS, these databases significantly enhance compound annotation accuracy and expand our understanding of broccoli’s complex volatilome [6,7]. Ultimately, they support innovations in broccoli breeding, postharvest processing, and consumption, contributing to improved crop quality and potential health benefits for consumers.

In this study, we aimed to construct a curated broccoli VOC database by comprehensively profiling the volatile metabolite composition across multiple broccoli cultivars. SPME coupled with GC-HRMS was employed using both EI and CI modes. This dual-ionization strategy enhances structural elucidation and expands metabolite coverage, providing a robust foundation for future efforts in cultivar differentiation, quality assessment, and metabolomics-guided breeding.

## 2. Results

### 2.1. Method Development

To construct a comprehensive volatile metabolite database for broccoli florets, it was first necessary to establish a standardized sample preparation and analytical protocol. An initial evaluation was performed using two capillary GC columns: TG-5 SilMS (30 m × 0.25 mm × 0.25 µm; Thermo Fisher Scientific, Waltham, MA, USA) and TG-5 MS (30 m × 0.25 mm × 0.25 µm; Thermo Fisher Scientific, Waltham, MA, USA) was conducted to optimize chromatographic separation and detection sensitivity. Among the tested columns, the TG-5 SilMS capillary column exhibited better overall performance, generating stronger signal intensities and a higher number of detectable peaks. Based on these results, the TG-5 SilMS column was selected for all subsequent analyses.

We then assessed the effects of key sample preparation parameters, including tissue state (fresh vs. freeze-dried), sample amount, extraction temperature, and extraction duration. Comparison of total ion chromatograms (TICs) between fresh and freeze-dried broccoli samples revealed similar profiles and signal intensities, suggesting that freeze-drying did not significantly alter the volatile composition. Consequently, freeze-drying was adopted as the standard sample preservation method due to its practicality and enhanced sample stability.

For volatile extraction, 200 mg of freeze-dried broccoli tissue was placed in a sealed headspace vial and pre-equilibrated in a 60 °C water bath for 15 min. Extraction was then carried out at 60 °C for 45 min using SPME, enabling efficient capture of volatile constituents.

In the preliminary phase of the study, both EI and CI modes were evaluated to assess their effectiveness in detecting volatile compounds from broccoli florets. EI produced higher signal intensities and a greater number of detectable peaks, offering rich fragmentation patterns that facilitated spectral matching. However, extensive fragmentation frequently obscured or eliminated the molecular ion, complicating the determination of molecular weights for unknown compounds. In contrast, CI generated fewer peaks and lower overall signal intensities but consistently provided intact molecular ions, which are essential for accurate compound identification.

By integrating data acquired from both EI and CI modes, we significantly improved the confidence and coverage of metabolite identification. The complementary strengths of these ionization techniques enhanced the robustness and reliability of volatile compound profiling, supporting more comprehensive characterization of broccoli’s volatilome.

### 2.2. n-Alkanes (C_8_–C_40_) and n-Nonyl Acetate (IS) Analysis

n-Alkanes (C_8_–C_40_) are widely employed as retention index (RI) calibration standards in GC-MS analysis due to their well-characterized and predictable retention behavior. In this study, a standard mixture of n-alkanes ranging from C_8_ to C_40_ was analyzed under identical chromatographic conditions as the broccoli floret samples, using both EI and CI modes. These straight-chain hydrocarbons provided a robust retention time reference, enabling the calculation of linear retention indices for unknown volatile compounds and thereby improving the accuracy and consistency of compound identification.

To further validate the analytical method, n-nonyl acetate was employed as an internal standard (IS). It was consistently detected at a retention time of 19.39 min, eluting between n-tridecane (C_13_) and n-tetradecane (C_14_). The calculated retention index (RI) for n-nonyl acetate was 1305, which closely aligns with the reported literature value of 1308 ± 4 [26]. This high level of agreement supports the reliability of the chromatographic system and confirms the accuracy of RI determination used throughout this study.

### 2.3. Database of Volatile Metabolites in Broccoli

**Figure 1:**
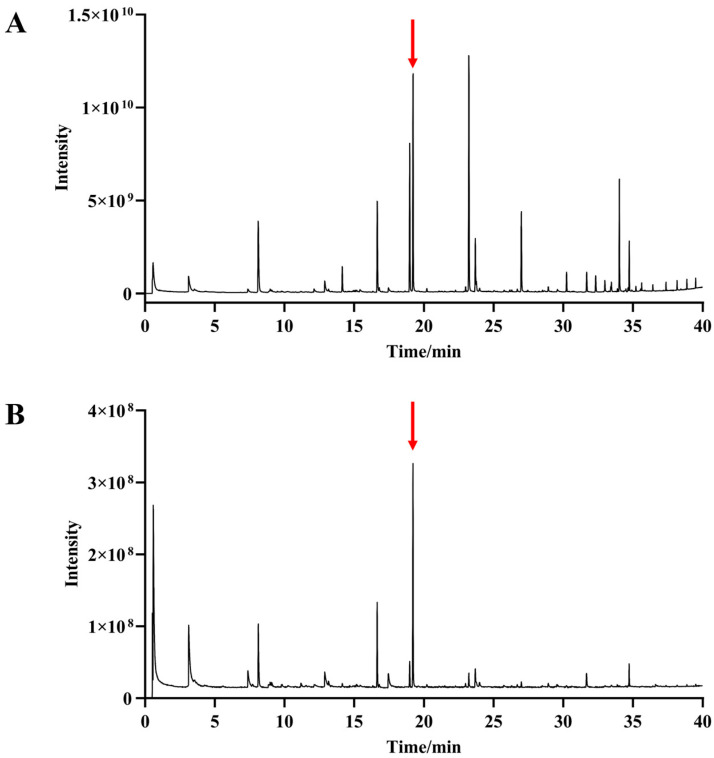
The TIC of Broccoli. (**A**), EI source. (**B**), CI source. The arrow in the figure indicates the IS, n-nonyl acetate.

To construct a comprehensive volatile metabolite database, a pooled reference sample was prepared using florets from 191 broccoli cultivars, collected and combined during December 2022 and January 2023. This pooled sample was designed to capture the broadest possible range of volatile compounds across diverse genetic backgrounds. Volatile metabolites were systematically analyzed using GC-MS, and the resulting TIC is shown in Figure 1. The internal standard, *n*-nonyl acetate, is clearly distinguishable in the TIC and was used to monitor instrument performance and ensure analytical stability throughout the experimental runs.

A total of 206 volatile metabolites were detected in broccoli florets, as summarized in Table S1. Of these, 37 compounds were further confirmed using synchronized GC-CI-MS analysis (Table 1), collectively accounting for approximately 47.79% of the TIC. The identified metabolites encompassed nine major chemical classes: esters, hydrocarbons, terpenoids, aldehydes, alcohols, ketones, heterocycles, ethers, and a miscellaneous group.

**Figure 2:**
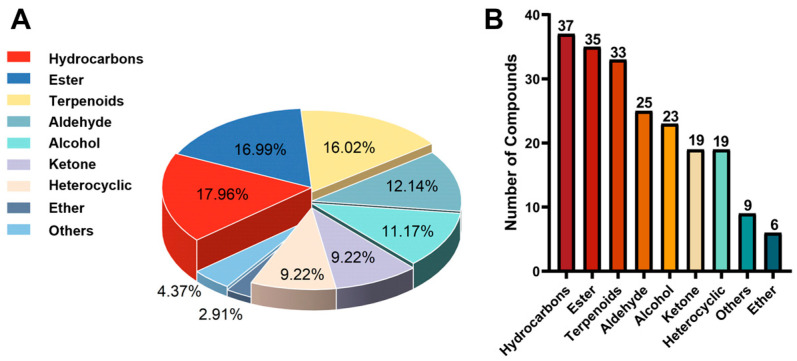
Volatile metabolites in broccoli florets. (**A**): chemical classes distribution, (**B**): number of compounds per chemical class.

Hydrocarbons represented the most abundant class, with 37 compounds (17.96%), followed by esters (35 compounds, 16.99%), terpenoids (33 compounds, 16.02%), aldehydes (25 compounds, 12.14%), and alcohols (23 compounds, 11.17%). Ketones and heterocycles each accounted for 19 compounds (9.22%), while ethers were the least represented, with 6 compounds (2.91%). The remaining 4.37% were classified as “other compounds,” which included sulfur-containing compounds, peroxides, epoxides, phenols, and nitriles (Figure 2).

Based on these results, a comprehensive database of volatile metabolites in broccoli was constructed, providing a valuable reference for future studies in flavor chemistry, cultivar differentiation, and Brassica metabolomics.

### 2.4. Volatile Metabolites in Different Varieties: Database Validation and Comparison

#### 2.4.1. Database Validation

**Figure 3:**
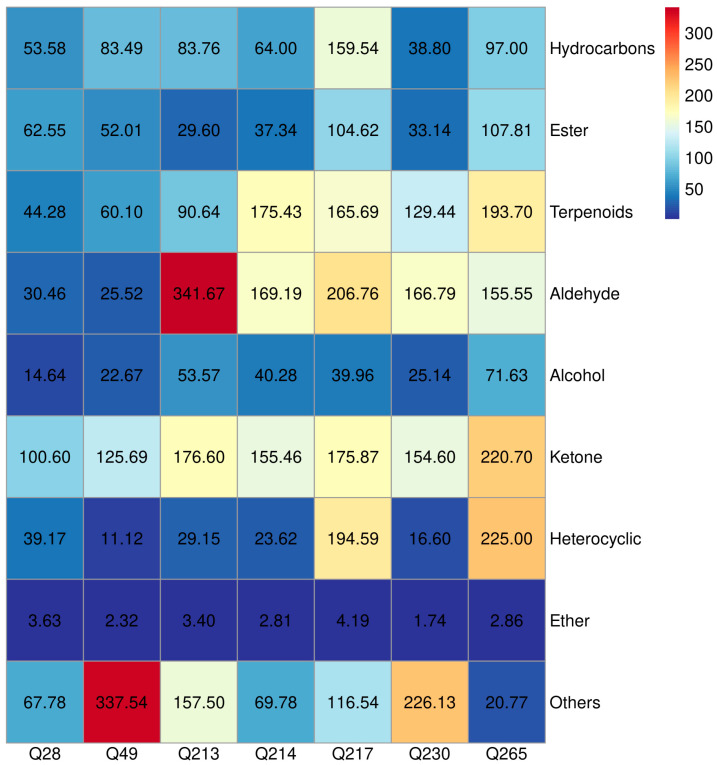
Contents of compound classes across seven broccoli cultivars.

To evaluate the reliability, reproducibility, and practical applicability of the constructed volatile metabolite database, broccoli florets from seven distinct cultivars were harvested in January 2025 under controlled field conditions. The collected samples were analyzed using the same SPME-GC-HRMS workflow, and metabolite identification was carried out by matching detected features against the curated database (Table S2). The distribution of compound classes across cultivars is illustrated in Figure 3.

Of the 206 volatile compounds included in the database, 187 (90.78%) were successfully detected in at least one of the seven cultivars, confirming the broad coverage and relevance of the compiled dataset. Nineteen compounds (9.22%) were not detected in any of the tested samples, potentially reflecting cultivar specificity, environmental dependence, or concentrations below the detection limit. Notably, 51 compounds (24.76%) were detected in only a single cultivar, highlighting the metabolic diversity among genotypes. In contrast, 38 compounds were consistently detected across all seven cultivars, suggesting the presence of a conserved core volatilome in broccoli.

#### 2.4.2. Esters

A total of 35 ester compounds were identified in the constructed volatile metabolite database. Among the seven broccoli cultivars analyzed, three esters were not detected in any sample, while nine were present in only a single cultivar, reflecting notable variation in ester distribution across genotypes. Only five esters—heptyl acetate, methyl caprate, 7,10,13-hexadecatrienoic acid methyl ester, methyl palmitate and methyl linolelaidate—were consistently detected in all seven cultivars, indicating a conserved subset of ester metabolites. Quantitative analysis revealed substantial differences in total ester content among the cultivars, with the highest concentration observed in variety Q265 (107.81 μg/kg) and the lowest in Q213 (29.60 μg/kg).

#### 2.4.3. Hydrocarbons

A total of 37 hydrocarbon compounds were identified in the constructed volatile metabolite database, encompassing alkanes, alkenes, and aromatic hydrocarbons. Among the seven broccoli cultivars analyzed, four hydrocarbons were not detected in any sample, while ten were found in only a single cultivar, indicating genotypic variability in hydrocarbon profiles. The highest total hydrocarbon content was observed in variety Q217 (159.54 μg/kg), with 3-octadecyne being the most abundant individual compound (123.63 μg/kg). In contrast, variety Q230 exhibited the lowest hydrocarbon content (38.80 μg/kg). Five hydrocarbons—cyclohexene, 2-ethenyl-1,3,3-trimethyl-, tridecane, pentadecane, 3-octadecyne, and nonacosane—were consistently detected across all seven cultivars, suggesting a conserved subset of hydrocarbon metabolites in broccoli.

#### 2.4.4. Terpenoids

Terpenoids are important secondary metabolites in Brassicaceae vegetables, including broccoli and cabbage, where they contribute to plant defense against herbivores, pathogens, and microbial stress [8]. In this study, terpenoids represented 16.02% of all volatile compounds identified in the constructed database. Among the seven broccoli cultivars analyzed, one terpenoid was not detected in any sample, while three were detected in only a single cultivar, indicating some degree of cultivar specificity.

The highest total terpenoid content was observed in variety Q265 (193.70 μg/kg), while isopimara-9(11),15-diene was the most abundant individual terpenoid in variety Q265 (89.69 μg/kg). In contrast, variety Q28 exhibited the lowest terpenoid concentration (44.28 μg/kg). Six terpenoids—citral, geosmin, β-ionone epoxide, cembrene, trachylobane, and isopimara-9(11),15-diene—were consistently detected across all seven cultivars. Notably, isopimara-9(11),15-diene exhibited relatively high concentrations in most varieties, suggesting its potential role as a core terpenoid component in broccoli.

#### 2.4.5. Aldehydes

A total of 25 aldehyde compounds were identified in the volatile metabolite database, accounting for 12.14% of the total detected compounds. Among the seven broccoli cultivars analyzed, two aldehydes were not detected in any sample, while nine were detected in only a single cultivar, suggesting a degree of cultivar-specific variation. The highest total aldehyde content was observed in variety Q213 (341.67 μg/kg), with pentanal being the most abundant (229.01 μg/kg). In contrast, variety Q49 exhibited the lowest aldehyde concentration (25.52 μg/kg). Four aldehydes—4-ethyl-benzaldehyde, 10-undecenal, 2-undecenal, and tetradecanal—were consistently detected across all seven cultivars, indicating their widespread presence in broccoli volatilomes. Notably, pentanal and (*E*)-2-hexenal were found at relatively high concentrations in most cultivars, except Q28 and Q49.

#### 2.4.6. Alcohols

A total of 23 alcohol compounds were identified, comprising 11.17% of the volatile metabolite database. Of these, two were not detected in any of the tested cultivars, and five were detected in only a single cultivar. The highest alcohol content was recorded in variety Q265 (71.63 μg/kg), with 5-methoxytryptophol as the dominant alcohol (40.09 μg/kg). In contrast, variety Q28 exhibited the lowest alcohol concentration (14.64 μg/kg). Four alcohols—3-octanol, 5-hepten-2-ol, 6-methyl-, 1-tetradecanol, and (*E*)-7-tetradecenol—were consistently detected across all cultivars. Among these, 3-octanol and (*E*)-7-tetradecenol were present at relatively high concentrations in all varieties. Additionally, 1-pentanol was notably more abundant in Q213 (13.17 μg/kg) and Q214 (8.07 μg/kg), suggesting potential cultivar-specific enrichment.

#### 2.4.7. Ketones

A total of 19 ketone compounds were identified in the volatile metabolite database, representing 9.22% of all detected metabolites. Among the seven broccoli cultivars analyzed, one ketone was not detected in any sample, while five were present in only a single cultivar. The highest total ketone content was observed in variety Q265 (220.70 μg/kg), whereas the lowest was found in Q28 (100.60 μg/kg). Eight ketones were consistently detected across all cultivars: 2,3-octanedione, 1-octen-3-one, (3*E*,5*E*)-3,5-octadien-2-one, 11-oxatetracyclo[5.3.2.0(2,7).0(2,8)]dodecan-9-one, trans-β-ionone, damascenone, β-damascenone, and nootkatone. Notably, trans-β-ionone was the most abundant ketone across all cultivars, accounting for over 55% of the total ketone content in each variety.

#### 2.4.8. Heterocyclic Compounds

Nineteen heterocyclic compounds were also identified, representing 9.22% of the total detected volatiles—matching the proportion of ketones. Among these, three compounds were not detected in any of the seven cultivars, while five were present in only one cultivar, indicating limited distribution. Varieties Q265 and Q217 exhibited the highest heterocyclic content, at 225.00 μg/kg and 194.59 μg/kg, respectively. In both cases, 6-methoxyquinoline-N-oxide was the dominant constituent, comprising 73.34% and 60.48% of the total heterocyclic content, respectively. In contrast, Q49 displayed the lowest heterocyclic concentration (11.12 μg/kg). Three heterocyclic compounds—2-amylfuran, 7-oxabicyclo[4.1.0]heptane, 3-oxiranyl-, and 3-methylindole—were consistently detected across all seven cultivars, suggesting a conserved presence within the broccoli volatilome.

#### 2.4.9. Ethers

Ethers were the least represented compound class in the volatile metabolite database, with only six compounds identified, accounting for 2.91% of the total detected volatiles. Among the seven broccoli cultivars, one ether was not detected in any sample, and two were found in only a single cultivar. Notably, only one ether compound was consistently detected across all cultivars, suggesting limited or cultivar-specific occurrence. Ether concentrations were uniformly low across all varieties (each < 5 μg/kg), with the highest content observed in variety Q217 (4.19 μg/kg), primarily attributed to 4-methylguaiacol (3.59 μg/kg). In contrast, Q230 exhibited the lowest ether content (1.74 μg/kg).

#### 2.4.10. Other Compounds

A total of nine compounds were classified as “other compounds,” including sulfur-containing compounds, peroxides, epoxides, and nitriles. Among these, two were not detected in any of the tested cultivars, and three were present in only one cultivar. Two compounds—decahydro-2,7a-dimethylnaphth[1,2-b]oxirene (an epoxide) and dimethyl trisulfide (a sulfur-containing compound)—were consistently detected across all seven cultivars. Dimethyl trisulfide was the most abundant compound in this category and the dominant sulfur-containing metabolite across all varieties, with the highest concentration observed in Q49 (337.38 μg/kg).

#### 2.4.11. Principal Component Analysis (PCA)

**Figure 4:**
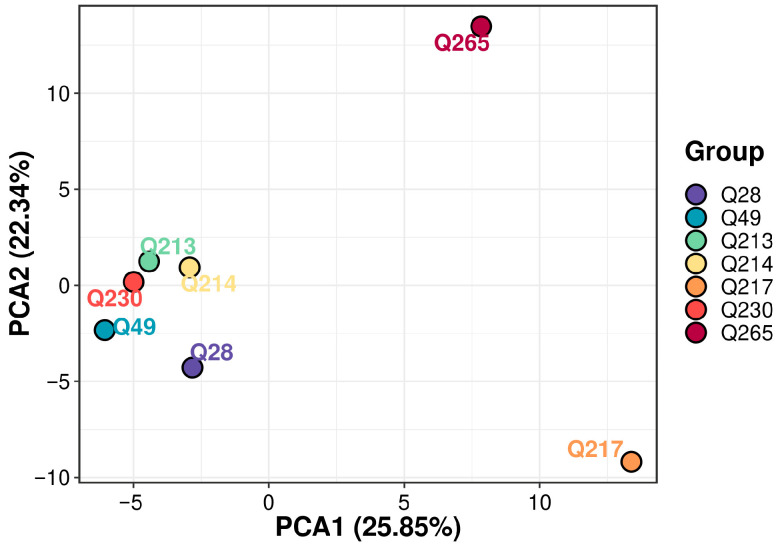
Principal component analysis of metabolites in Seven Broccoli Varieties.

PCA was conducted to assess the variation and underlying patterns in the VOC profiles of the seven selected broccoli cultivars. The first two principal components, PC1 and PC2, accounted for 25.85% and 22.34% of the total variance, respectively (Figure 3). The PCA score plot (Figure 4) revealed clear separation of cultivars Q217 and Q265 from the remaining five varieties, indicating distinct differences in both the composition and relative abundance of VOCs. This separation highlights the unique volatile profiles of Q217 and Q265, which may be attributed to cultivar-specific metabolic traits or genetic background.

## 3. Discussion

### 3.1. Fresh and Freeze-Dried Sample Preparation for Volatile Profiling

Sample preparation is a critical determinant in the accurate detection and quantification of VOCs in plant metabolomic studies. In this work, we assessed the impact of sample state—fresh versus freeze-dried—on the VOC profiles of broccoli florets using GC-MS. TICs obtained from both sample types exhibited strong similarity in peak shape, signal intensity, and compound distribution, indicating that freeze-drying had minimal influence on the overall volatile composition.

Fresh tissue is often regarded as more reflective of the in vivo metabolic state, particularly for compounds with high volatility or those formed via active enzymatic processes. However, high water content can hinder extraction efficiency and introduce variability due to inconsistent moisture levels and enzymatic changes after harvest [27]. In contrast, freeze-dried samples offer several practical and analytical benefits, including improved sample homogeneity, better long-term stability, and enhanced throughput for large-scale studies [28,29]. Lyophilization also concentrates volatiles by removing water, thereby minimizing matrix effects during extraction.

While concerns remain about potential loss of highly labile or low-boiling-point compounds during freeze-drying, our findings demonstrate that the majority of broccoli volatiles were preserved, with no significant reduction in TIC intensity or compound diversity. This aligns with previous studies reporting the effective retention of plant volatiles under proper lyophilization and storage conditions [30,31].

Given these advantages, freeze-dried samples were selected as the standard preparation method for this study, ensuring reproducibility and practicality for comprehensive profiling and database development. Nonetheless, for studies targeting ultra-volatile or transient compounds, or those requiring real-time analysis, fresh samples may still be more suitable.

### 3.2. Pooled-Sample Strategy for Comprehensive Volatile Metabolite Database Construction

To construct a comprehensive and representative volatile metabolite database for broccoli, a pooled-sample approach was employed. Equal portions of florets from 191 distinct broccoli cultivars were combined to generate a composite sample that reflects the broad chemical diversity across genotypes. This strategy ensures inclusion of both ubiquitous metabolites and those that may be unique to specific cultivars, thereby enhancing the database’s generalizability and utility. Pooling reduces cultivar-specific variability and allows for the detection of a wider array of compounds, including those present at low abundance or only in trace amounts in individual varieties [32,33]. Moreover, this approach minimizes biological noise and enhances compound detectability by amplifying signals from rare or cultivar-specific volatiles.

The composite sample was analyzed using GC-HRMS under both EI and CI modes, facilitating the development of a robust, high-confidence reference database for volatile metabolite profiling in broccoli and related *Brassica* species.

The utility of the pooled-sample-based database was validated using individually harvested florets from seven distinct broccoli cultivars. Among the 206 volatile compounds recorded in the database, 187 (90.78%) were detected in at least one of the tested cultivars. These results confirm that the pooled approach effectively captured both core and variable metabolites, supporting its effectiveness in representing the metabolic diversity of broccoli. The successful identification of compounds across diverse genotypes further underscores the reliability and broad applicability of this reference database in future metabolomic studies [34].

### 3.3. Validation and Applicability of the Volatile Metabolite Database

To evaluate the reliability, reproducibility, and practical utility of the constructed volatile metabolite database, florets from seven genetically distinct broccoli cultivars were analyzed using the standardized GC-HRMS workflow. Metabolite identification was performed via spectral matching against the curated database. Of the 206 volatile compounds cataloged, 187 (90.78%) were detected in at least one cultivar, highlighting the comprehensive coverage and biological relevance of the database to the broccoli volatilome. The remaining 19 compounds (9.22%) were not observed in any of the tested samples, potentially due to cultivar specificity, inducible biosynthesis under specific environmental conditions (e.g., biotic or abiotic stress), or concentrations below the analytical detection limit.

Interestingly, 51 compounds (24.76%) were uniquely detected in a single cultivar, reflecting substantial metabolic diversity among genotypes. In contrast, 38 volatiles were consistently detected across all seven cultivars, indicating the presence of a conserved core volatilome in broccoli. These findings demonstrate that the database effectively captures both ubiquitous and genotype-specific volatile metabolites, providing a valuable tool for comprehensive metabolic profiling. Its high coverage and discriminatory power position it as a robust platform for diverse applications in *Brassica* metabolomics, including cultivar differentiation, quality assessment, sensory trait selection, and studies on plant—environment interactions.

### 3.4. VOC Profile Differences Among 7 Varieties

Significant VOC profile differences were observed in the Q217 and Q265 samples compared to the other groups. These differences are likely due to cultivar-specific variations in metabolite composition, which can arise from genetic factors, developmental stage, or subtle differences in growth conditions. These findings highlight the impact of genetic diversity on shaping broccoli volatile profiles and suggest that Q217 and Q265 may possess unique sensory characteristics or bioactive potential.

In particular, Q217 accumulated higher levels of esters, ketones, hydrocarbons, and terpenes, whereas Q265 produced elevated levels of esters, heterocyclic compounds, ketones, and terpenes. Esters typically arise from the reaction of alcohols with acyl-CoA derivatives via alcohol acyltransferases, and their increased abundance in Q217 and Q265 may contribute fruity or floral notes that distinguish these cultivars. Heterocyclic compounds, which can originate from Maillard-type reactions or amino acid degradation pathways, may be more prominent in Q265 and contribute to roasted or sulfur-like aromas. Ketones, often derived from lipid oxidation or branched-chain amino acid catabolism, were also more abundant in both cultivars and may explain their separation along PCA1. Hydrocarbons, commonly formed via fatty acid decarboxylation, and terpenes, synthesized through the mevalonate or MEP pathways, were also altered, reflecting genetic regulation of primary and secondary metabolism.

Taken together, the distinct clustering of Q217 and Q265 likely reflects the combined effects of differential activity in ester biosynthesis, amino acid and lipid degradation, fatty acid metabolism, and terpene synthesis. These results underscore how genetic and biochemical diversity among broccoli cultivars can strongly influence the balance of volatile classes, leading to unique sensory attributes and potential bioactivity.

## 4. Materials and Methods

### 4.1. Chemicals and Reagents

n-Nonyl acetate, n-Alkanes (C8–C40) and LC grade methanol were obtained from Sigma-Aldrich (St. Louis, MO, USA). All other chemicals were of analytical reagent grade and were purchased from commercial suppliers.

### 4.2. Plant Materials

To obtain more comprehensive coverage of volatile metabolites and improve detection efficiency, a pooled sample strategy was employed. Broccoli florets from 191 cultivars were collected in December 2022 and January 2023. All cultivars were grown under standardized management practices at the experimental base of the Wenzhou Academy of Agricultural Sciences in Wenzhou, China (120.51° E, 28.06° N, elevation 10 m). Broccoli florets were harvested at the commercial maturity stage, promptly frozen in liquid nitrogen, and subsequently stored at −80 °C prior to freeze-drying. Approximately 5 g of florets from each cultivar were freeze-dried using a vacuum freeze dryer (LGJ-12, Songyuan Huaxing Technology Development Co., Ltd., Beijing, China) at a condenser temperature of −60 °C and a pressure of 5 Pa for 48 h. The freeze-dried florets were then ground into a fine powder using a freeze grinding machine with a 50 mL container (JXFSTPRP-CLN, Shanghai Jingxin Industrial Development Co., Ltd., Shanghai, China). This procedure was consistent across all samples to minimize pre-analytical variation in metabolite profiles. For pooled sample, 50 mg of dried material per cultivar was combined and further homogenized using the same grinder to generate a pooled sample. Six pooled replicate samples were prepared in this manner and stored at −80 °C until analysis. To validate the database, floret samples from seven (Table 2, Supplementary Figure S1) of the 191 cultivars were selected in January 2025, freeze-dried, and analyzed individually.

### 4.3. SPME Extraction of Volatile Compounds

A total of 200 mg of freeze-dried broccoli sample was weighed and transferred into a 20 mL headspace vial (P/N 20-HSV, Thermo Fisher Scientific, Waltham, MA, USA). Then, 1 μL of *n*-nonyl acetate (4.59 μmol/mL) was added as an internal standard, and the vial was tightly sealed. The sealed vial was equilibrated in a 60 °C water bath for 15 min. Prior to use, the SPME fiber (100 μm PDMS, Supelco, Bellefonte, PA, USA) was preconditioned in the GC injection port for 5 min. After equilibration, the fiber was inserted into the vial headspace and exposed at 60 °C for 45 min to extract volatile compounds. Following extraction, the fiber was immediately transferred to the GC injection port and desorbed at 250 °C for 5 min. The extracted volatiles were then analyzed by GC-MS for qualitative identification. Double blank controls (without plant material and internal standard) and blank controls (with internal standard only) were included to eliminate background signals and potential extraction artifacts.

### 4.4. GC–Orbitrap MS Analysis of Volatile Metabolites

Volatile metabolites in broccoli were analyzed using a Trace 1310 gas chromatograph coupled with a Q Exactive GC Orbitrap high-resolution mass spectrometer (Thermo Fisher Scientific, Waltham, MA, USA), equipped with a TG-5 SilMS capillary column (30 m × 0.25 mm × 0.25 μm). The inlet temperature is 250 °C, equipped with 1.2 mm inlet liner (P/N 453A1335, Thermo Fisher Scientific, Waltham, MA, USA) for SPME analyses. Gas chromatographic separation was performed using the following oven temperature program: initial temperature of 40 °C (held for 2 min), ramped at 5 °C/min to 180 °C (held for 2 min), then increased at 15 °C/min to 320 °C (held for 8 min). To enhance compound identification, both EI and CI modes were employed.

In EI mode, the ionization energy was set to 70 eV with full-scan acquisition over an *m*/*z* range of 50–500. The total run time was 50 min with no solvent delay. The injector, transfer line, and ion source temperatures were 250 °C, 260 °C, and 280 °C, respectively. Samples were injected in splitless mode, and helium served as the carrier gas at a constant flow rate of 1 mL/min.

In CI mode, the scan range was *m*/*z* 30–500, with the same 50 min run time and no solvent delay. The transfer line and CI ion source temperatures were set at 260 °C and 200 °C, respectively. Methane was used as the reagent gas at a flow rate of 1.5 mL/min. The injection conditions and carrier gas settings matched those used in EI mode.

### 4.5. Data Processing

Compound identification was primarily performed using the NIST 2017 mass spectral database. For analytes detected via EI, a two-step identification approach was applied. First, mass spectra were matched against several high-resolution libraries, including the Flavor & Off-Flavor HRMS Library, for preliminary annotation. Identification was then confirmed by comparing calculated RIs with values from the NIST RI database.

To ensure data quality, TICs were evaluated for baseline stability, peak shape, and signal-to-noise ratio using Xcalibur software (v4.5.18). Prior to identification, deconvolution was conducted to enhance resolution, and the data were processed in TraceFinder (v5.1) using the following parameters: mass deviation ≤ 5 ppm, ion signal-to-noise threshold ≥ 3, TIC threshold ≥ 500,000, and fragment ion overlap match ≥ 98%. For library matching, filters included a total score ≥ 90, similarity index ≥ 650, high-resolution filteringscore ≥ 90, and enabled fragment annotation. RI calibration was conducted using a standard *n*-alkane series, and background signals were removed via blank subtraction before peak alignment. Final deconvoluted results were exported to Excel for statistical analysis. Relative metabolite levels were calculated using the peak area normalization method, expressed as the ratio to the internal standard, following the protocol described by Majithia (2021) [35].

Compound identification was further supported by assigning carbon numbers based on *n*-alkane retention times and integrating mass spectral matches, retention indices, and diagnostic fragment ions. Identified compounds were reported with their name, retention time, CAS number, and molecular formula. All raw and processed data were archived for full traceability. Quality control procedures and optimized spectral matching criteria were applied throughout to ensure identification accuracy and reproducibility.

For compounds detected via CI, data were analyzed using the Xcalibur Qual Browser, Thermo Fisher Scientific, Waltham, MA, USA. TICs and high-resolution mass spectra were manually inspected, and protonated molecular ions ([M + H]^+^) were determined with five-decimal precision. Molecular formulas were inferred based on exact mass and carbon number information from *n*-alkane standards. Results from EI analysis were used to support and cross-validate CI-based identifications.

### 4.6. Statistical Analysis

All experimental data were analyzed using Microsoft Excel 2021 and GraphPad Prism version 8.3.0. Principal component analysis and heat map analysis were conducted using the OmicStudio platform (https://www.omicstudio.cn/tool, accessed on 15 July and 26 August 2025). Each experiment was performed with three independent biological replicates.

## 5. Conclusions

In this study, we developed a comprehensive volatile metabolite database for broccoli using SPME-GC-HRMS with dual ionization modes (EI and CI). A pooled sample derived from 191 broccoli cultivars was analyzed to maximize chemical diversity and minimize biological variability. A total of 206 volatile compounds spanning nine chemical classes were detected, 37 of which were confirmed via CI-based molecular ion information. The constructed database was validated using florets from seven distinct cultivars, where 90.78% of the compounds were successfully identified, and a conserved core set of 37 volatiles was observed. Chemical class-specific patterns and cultivar-level differences were further characterized using multivariate analysis. Additionally, freeze-dried sample preparation was validated as a reliable approach for large-scale volatile profiling. Overall, this robust and versatile database provides a valuable resource for future studies in *Brassica* metabolomics, enabling applications in cultivar differentiation, sensory trait improvement, and environmental response analysis.

## Figures and Tables

**Table 1:** 37 compounds confirmed by both EI and CI modes.

No *	Compound Name	Retention Time	Reference *m*/*z*	Avg Peak Area	Formula	CAS No.	Avg Calculated RI	Library RI	Classes
2	2-Hexenal, (*E*)-	3.11	41.0386	551,060,469	C_6_H_10_O	6728-26-3	N/C **		Aldehyde
5	Thiophene, 3-ethyl-	3.55	97.0106	21,633,341	C_6_H_8_S	1795-01-3	N/C		Heterocyclic
6	Allylacetone	3.64	43.0178	50,094,456	C_6_H_10_O	109-49-9	N/C		Ketone
7	*p*-Xylene	3.78	91.0542	20,357,362	C_8_H_10_	106-42-3	N/C		Hydrocarbons
12	Allyl Isothiocyanate	4.67	99.0137	2,235,272	C_4_H_5_NS	57-06-7	N/C		Others
13	*o*-Xylene	4.85	91.0542	10,171,454	C_8_H_10_	95-47-6	N/C		Hydrocarbons
14	Benzocyclobutene	4.87	104.0621	25,872,768	C_8_H_8_	694-87-1	N/C		Hydrocarbons
17	Caproic acid methyl ester	6.56	74.0362	7,124,719	C_7_H_14_O_2_	106-70-7	863	925	Ester
18	7-Azabicyclo[4.1.0] heptane, 2-methyl-	7.39	96.0808	1,110,790	C_7_H_13_N	55903-15-6	896	948	Heterocyclic
20	*n*-propylbenzene	7.52	91.0542	1,887,762	C_9_H_12_	103-65-1	901	953	Hydrocarbons
21	7-Azabicyclo[4.1.0] heptane, 1-methyl-	7.71	82.0651	6,019,497	C_7_H_13_N	25022-25-7	908	961	Heterocyclic
25	Heptanonitrile	8.98	41.0386	113,477,389	C_7_H_13_N	629-08-3	951	988	Others
26	2,3-Octanedione	8.99	43.0178	81,773,661	C_8_H_14_O_2_	585-25-1	951	984	Ketone
27	2-Amylfuran	9.06	81.0335	47,171,653	C_9_H_14_O	3777-69-3	954	993	Heterocyclic
28	Cyclopentane, 1-methyl-3-(2-methyl-1-propenyl)-	9.26	81.0699	15,627,543	C_10_H_18_	75873-01-7	960	972	Hydrocarbons
37	d-Limonene	10.44	68.0621	217,256,255	C_10_H_16_	5989-27-5	1001	1018	Terpenoids
41	2,2-Dimethyl-1-aza-spiro[2.4]heptane	11.2	110.0964	584,869	C_8_H_15_N	N/A ***	1023	1034	Heterocyclic
44	3-[(*E*)-3-Methyl-1-butenyl]-1-cyclohexene	12.03	79.0542	6,086,431	C_11_H_18_	56030-49-0	1054	1104	Hydrocarbons
45	(3*E*,5*E*)-3,5-Octadien-2-one	12.10	95.0491	187,208,595	C_8_H_12_O	30086-02-3	1056	1073	Ketone
52	Cyclohexene, 2-ethenyl-1,3,3-trimethyl-	13.08	135.1168	8,886,180	C_11_H_18_	5293-90-3	1089	1105	Hydrocarbons
74	Naphthalene	15.76	128.0621	1,425,023	C_10_H_8_	91-20-3	1181	1182	Hydrocarbons
82	Dimethyltetrasulfane	16.65	157.9347	831,336,230	C_2_H_6_S_4_	5756-24-1	1315	1234	Others
84	Citral	16.79	69.0699	311,692,396	C_10_H_16_O	5392-40-5	1217	1276	Terpenoids
93	1,7-Octadiene-3,6-diol, 2,6-dimethyl-	18.15	67.0542	2,553,586	C_10_H_18_O_2_	51276-33-6	1267	1273	Terpenoids
95	1-Methoxyindole	18.32	117.0573	50,967,860	C_9_H_9_NO	54698-11-2	1273	1218	Heterocyclic
102	Methyl caprate	19.55	74.0362	122,090,959	C_11_H_22_O_2_	110-42-9	1318	1325	Ester
138	trans-β-lonone	23.68	177.1274	1,314,889,389	C_13_H_20_O	79-77-6	1481	1486	Ketone
148	Benzene, 1,4-bis(1-formylethyl)-	25.73	161.0961	13,325,826	C_12_H_14_O_2_	N/A ***	1568	1553	Aldehyde
157	Tetradecanal	26.68	57.0335	282,159,980	C_14_H_28_O	124-25-4	1609	1613	Aldehyde
164	7-Methoxy-4-quinolinol	28.54	175.0628	2,991,165,529	C_10_H_9_NO_2_	82121-05-9	1694	1635	Heterocyclic
166	1-Heptadecyne	28.93	81.0699	84,475,533	C_17_H_32_	26186-00-5	1712	1709	Hydrocarbons
169	Arvelexin	29.54	171.0553	846,914	C_11_H_10_N_2_O	4837-74-5	1740	1798	Heterocyclic
178	3-Octadecyne	31.67	67.0542	547,120,892	C_18_H_34_	61886-64-4	1838	1828	Hydrocarbons
180	7,10,13-Hexadecatrienoic acid, methyl ester	33.01	79.0542	58,497,194	C_17_H_28_O_2_	56554-30-4	1896	1902	Ester
181	Methyl palmitate	33.45	74.0362	26,907,298	C_17_H_34_O_2_	112-39-0	1924	1926	Ester
197	Methyl Linolenate	35.62	79.0542	86,055,764	C_19_H_32_O_2_	301-00-8	2096	2098	Ester
201	3,4′-Isopropylidenediphenol	36.62	213.0910	156,994,944	C_15_H_16_O_2_	46765-25-7	2211	2173	Others

*: Compound number same as that in Table S1. **: Compound eluted ahead octane, Calculated RI is not calculatable marked as N/C. ***: Compounds without assigned CAS registry numbers are indicated as N/A.

**Table 2:** Seven cultivars harvested in January 2025.

No.	Cultivar	Provider
Q28	Yacui 60	Shouguang Syngenta Seed Co., Ltd., Shouguang, China
Q49	Zheqing 161	Zhejiang Academy of Agricultural Sciences, Hangzhou, China
Q213	W10	Wenzhou Academy of Agricultural Sciences, Wenzhou, China
Q214	W11	Wenzhou Academy of Agricultural Sciences, Wenzhou, China
Q217	W14	Wenzhou Academy of Agricultural Sciences, Wenzhou, China
Q230	Wancui 2	Shouguang Syngenta Seed Co., Ltd., Shouguang, China
Q265	Zhongqing 518	Institute of vegetables and flowers, Chinese academy of agricultural Sciences, Beijing, China

## Data Availability

The data presented in this study are available in this article and Supplementary Materials.

## Supplementary Materials

The following supporting information can be downloaded at: https://www.mdpi.com/article/10.3390/molecules30183781/s1, Table S1: 206 volatile metabolites were detected in broccoli florets; Table S2: Contents of volatile metabolites across seven broccoli cultivars. Table S3: Information on 191 broccoli cultivars. Figure S1. The collected samples of broccoli floret from 7 cultivars.

## Abbreviations

The following abbreviations are used in this manuscript:

CI	Chemical ionization
EI	Electron ionization
GC-MS	Gas chromatography–mass spectrometry
HRMS	High-resolution mass spectrometry
PCA	Principal component analysis
SD	Standard deviation
SPME	Solid-phase microextraction
TIC	Total ion chromatogram
VOC	Volatile organic compounds
