# Supplementary figures and images for: Dual-Ionization SPME-GC–HRMS Metabolomic Profiling of Broccoli Volatiles for the Construction of a Broccoli Metabolic Database
Source: Molecules. 2025 Sep 17;30(18):3781. doi: 10.3390/molecules30183781 (PMC12472610; doi:10.3390/molecules30183781)

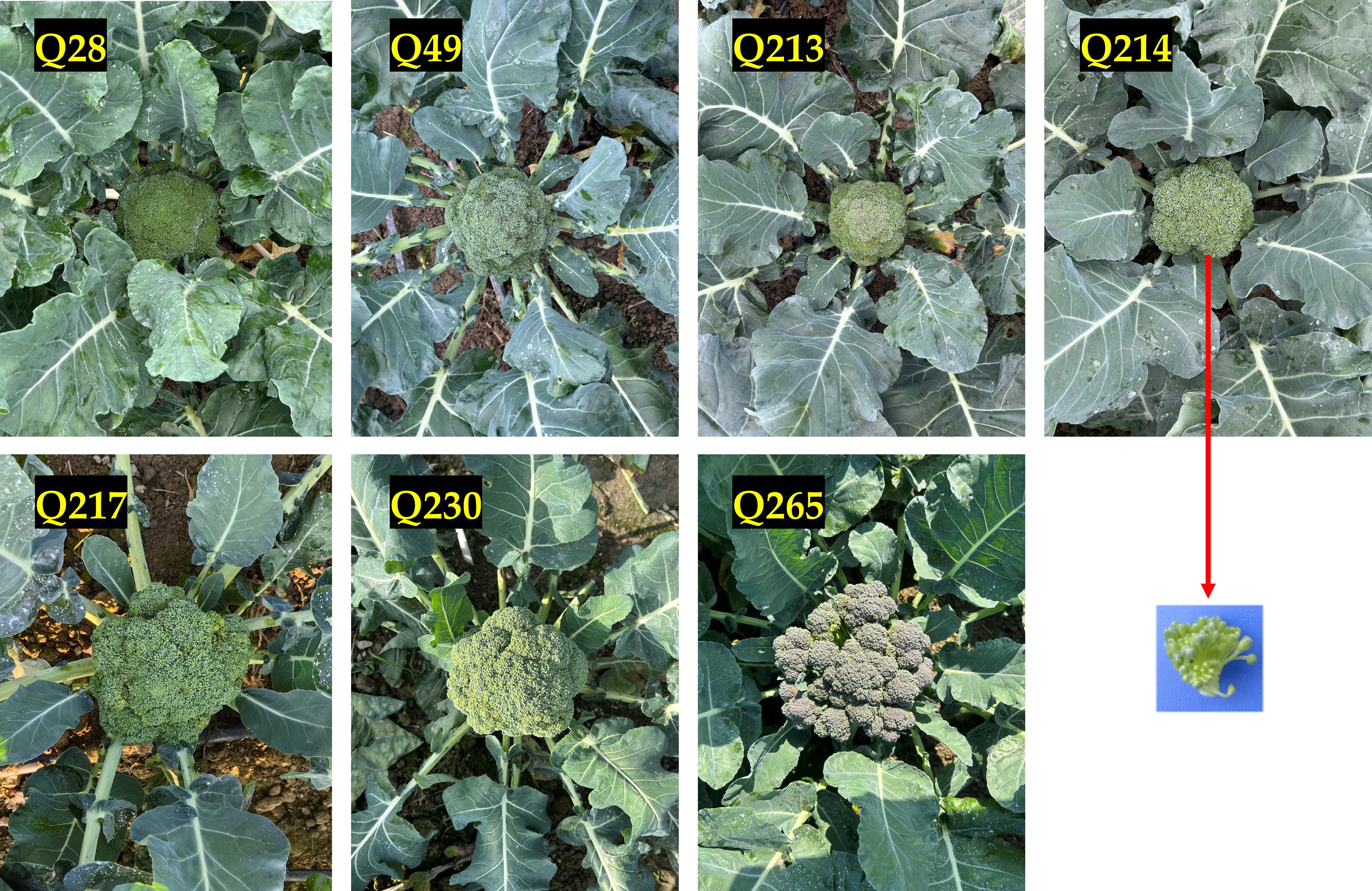

Supplement: Supplementary file 1 [file molecules-30-03781-s001.zip › Figure S1. The collected samples of broccoli floret from 7 genotypes.jpg]
